# Impact of *porcine circovirus type 2* on *porcine epidemic diarrhea virus* replication in the IPI-FX cell line depends on the order of infection

**DOI:** 10.3389/fmicb.2023.1162104

**Published:** 2023-03-30

**Authors:** Hao Zhang, Hongyan Shi, Yanwu Wei, Da Shi, Mengxiang Cao, Jianbo Liu, Jianhang Liu, Liang Li, Changming Liu, Li Feng, Liping Huang

**Affiliations:** Division of Swine Digestive System Infectious Diseases, State Key Laboratory for Animal Disease Control and Prevention, Harbin Veterinary Research Institute, Chinese Academy of Agricultural Sciences, Harbin, China

**Keywords:** porcine circovirus type 2, porcine epidemic diarrhea virus, IPI-FX cells, IPEC-J2 cells, co-infection

## Abstract

**Introduction:**

A study in 2006 showed that the clinical course of PEDV disease was markedly aggravated by transplacental infection of PCV2. Therefore, we investigated whether the small intestine supports PCV2 replication and the effect of PCV2 infection on PEDV replication in epithelial cells *in vitro*.

**Methods:**

To confirm the intestinal tropism of PCV2, the viral loads in the small-intestinal tissues after PCV2 infection were determined with virus titration, and the viral titers in the infected pig jejunum, ileum, ileocecal valve, and colon were 10^4.86^, 10^4.09^, 10^2.52^, and 10^2.35^ TCID_50_/g, respectively. We then determined the propagation characteristics of PCV2 in ileal epithelial cells (IPI-FX) and jejunal epithelial cells (IPEC-J2) with an immunoperoxidase monolayer assay, virus titration, and an immunofluorescence assay. Both IPI-FX and IPEC-J2 cells supported the replication of PCV2, with titers of 10^5.5^ and 10^5.0^ TCID_50_/ml, respectively. We established an infection model of PCV2 and PEDV in IPI-FX cells and found that PEDV and PCV2 infected the cells individually and together. The effects of PCV2 infection on PEDV replication were determined with reverse transcription–quantitative PCR (qPCR), western blotting, and virus titration. When PCV2 infected IPI-FX cells before PEDV, PCV2 significantly inhibited the replication of PEDV in a dose- and time-dependent manner and that the mRNAs of IFN-β, TNF-α, IL1β, and OASL were downregulated (detected with qPCR). Surprisingly, when IPI-FX cells were co-infected with PCV2 and PEDV, PCV2 promoted the replication of PEDV, the expression of the host IFN-β, TNF-α, IL1β, and OASL mRNAs was upregulated.

**Discussion:**

These findings demonstrate that the co-infection of IPI-FX cells with PCV2 and PEDV represents an excellent *in vitro* model in which to investigate their combined pathogenic mechanisms.

## 1. Introduction

*Porcine circovirus type 2* (PCV2), a nonenveloped virus with a single-stranded circular DNA genome of about 1.7 kb (Allan et al., 1998), belongs to the genus *Circovirus* in the family *Circoviridae* and is the smallest known DNA virus to infect mammals. As other ssDNA virus, PCV2 is characterized by a highly evolutionary rate, leading to the emergence of variants with different biological features. Based on the latest classification scheme, PCV2 was divided into 8 genotypes (PCV2a-2 h). Among them, PCV2a was the most prevalent genotype in piglets in 1996–2005, after which PCV2b predominated. PCV2d was globally epidemic variant around 2012, perhaps driven by the worldwide use of PCV2 vaccines. In Asia, PCV2d, 2b and 2a are the top three epidemic strains, respectively, with PCV2d having a prevalence rate of more than 50% (Franzo and Segales, 2018). Because PCV2 is highly resistant to the environment (Lopez-Lorenzo et al., 2019), the body fluids of infected pigs, including oral and nasal fluids, feces, urine, and semen (Patterson and Opriessnig, 2010), are sources of infection. PCV2 is considered the main pathogen of porcine-circovirus-associated diseases (PCVAD) (Chae, 2005), including postweaning multisystemic wasting syndrome (PMWS), porcine respiratory disease complex, reproductive disorders, and porcine-circovirus-associated enteritis (Ramamoorthy and Meng, 2009). The main clinical manifestations and pathological changes in porcine-circovirus-associated enteritis are diarrhea and granulomatous enteritis with lymphocyte depletion and granulomatous inflammation in Peyer’s patches, accompanied by moderate-to-high amounts of PCV2 in the intestinal mucosa and Peyer’s patches (Kim et al., 2004). The distribution of PCV2 in the porcine intestine is currently unknown. However, although PCV2 can replicate in intestinal tissues, it is unclear whether small-intestinal epithelial cell lines support PCV2 replication.

*Porcine epidemic diarrhea virus* (PEDV) is a member of the genus *Coronavirus*, family *Coronaviridae*, and order *Nidovirales*. PEDV mainly infects piglets, and infection manifests as watery diarrhea and vomiting. It has a high infection rate and high lethality in newborn piglets. Necropsy reveals a thinned bowel wall containing pale yellow fluid (Madson et al., 2014). PEDV infects the jejunum and ileum (Kim and Chae, 2000), causing atrophy and the shedding of the intestinal villi. The target cells of PEDV *in vivo* in piglets are predominantly enterocytes, but infection can also be found in intestinal stem cells, proliferating cells, and goblet cells (Li et al., 2019).

In 2006, a retrospective study in South Korea showed that the co-infection rate of PCV2 and PEDV was 32.7% in 107 tissue samples infected with PEDV (Jung et al., 2006a). The testing of piglets with diarrhea showed that co-infection with PEDV and PCV2 was common in Hunan, Shanxi, Shaanxi, Henan, Sichuan, and other places in China, with co-infection rate ranging from 3.47 to 57.89% in different age groups (Guo et al., 2020; Ma et al., 2021). It has also been reported that PEDV causes more-serious histopathological changes in piglets already infected with PCV2 (Jung et al., 2006b). Therefore, the specific ways in which the two viruses interact after infection warrants further investigation.

In this study, the intestinal tissue tropism of PCV2 was determined by detecting PCV2 and its DNA in different intestinal segments from PCV2-infected piglets. More importantly, IPI-FX cells were established as an *in vitro* infection model to study the effects of PCV2 infection on PEDV replication and the cytokines it induces.

## 2. Materials and methods

### 2.1. Cells and viruses

PK15 cells (CCL-33) free of PCV1 and PCV2, Vero E6 cells (CRL-1586), IPEC-J2 cells (Rhoads et al., 1994), and IPI-FX cells (Wang et al., 2019) were grown in Dulbecco’s modified Eagle’s medium (DMEM; Invitrogen, Carlsbad, CA, United States) containing 10% heat-inactivated fetal bovine serum (FBS; Gibco, Grand Island, NY, United States). The PCV2a-LG strain (accession number: HM038034), PCV2b-MDJ strain (accession number: OL452025) and PCV2d-LNHC strain (accession number: OL452027) were propagated in PK15 cells (Yu et al., 2022), and the PEDV-SX1a strain was propagated in Vero E6 cells.

### 2.2. Intestinal tissue samples

Six four-week-old cross-bred piglets were confirmed as negative for PCV2, PEDV, *African swine fever virus* (ASFV), *Porcine reproductive and respiratory syndrome virus* (PRRSV), and pseudorabies virus (PRV) with PCR. All the piglets underwent a 3-day acclimatization period before inoculation. The piglets were then randomly assigned to two groups, with three piglets in each group: the PCV2 infection group and the mock-infection group. In the PCV2 infection group, the piglets were inoculated with PCV2d-LNHC by intranasal and intramuscular injection with 10^5.5^ 50% tissue culture infective doses (TCID_50_) per route, whereas the piglets in the mock-infected group were inoculated with an equal amount of DMEM as the control. The piglets in the infected and mock-infected groups were housed in different rooms. All the piglets were euthanized with xylazine and their jejunums, ileums, colons, and ileocecal valves were collected at 4 weeks post infection, and the contents were rinsed off. The collected tissues were ground to a 0.1 g/ml emulsion in DMEM and stored at −20°C.

### 2.3. Detection of PCV2 in intestinal tissue with virus titration and quantitative PCR (qPCR)

Virus titration was performed as previously described to determine the viral titers of PCV2 in the tissue samples described above (Yu et al., 2022). The sample emulsions were centrifuged at 872 × g for 10 min and sterilized with 0.22 μm filters. The filtered samples were serially diluted 10-fold with DMEM from 10^−1^ to 10^−5^, and the diluted samples (100 μl/well) were then added to four wells containing a 50% monolayer of PK15 cells. At the same time, four wells containing uninfected cells were used as the controls. The PK15 cells were incubated at 37°C under 5% CO_2_ for 1 h. The medium was then replaced with DMEM containing 2% FBS and incubated for 72 h at 37°C under 5% CO_2_. An immunoperoxidase monolayer assay (IPMA) was performed as described previously (Huang et al., 2011) with mouse anti-serum directed against the PCV2 Cap protein and horseradish-peroxidase-labeled rabbit anti-mouse IgG (H + L) (Sigma-Aldrich, St. Louis, MO, United States) as the primary and secondary antibodies, respectively. The viral titers of PCV2 in the samples were calculated with the Reed–Muench method.

QPCR with a pair of specific primers and a probe (Table 1) was used to determine the genome copies of PCV2 in the intestinal tissue samples from infected and mock-infected piglets. DNA was extracted from the intestinal tissue emulsions with blood/cell/tissue genomic DNA extraction kit (Tiangen, Beijing, China). All DNA extracts were tested for the presence of PCV2 DNA with qPCR targeting a conserved region of the genome, as described previously (Huang et al., 2016). The results were calculated as the mean of the logarithmic viral DNA copy number per gram of inguinal lymph node (log10 copies/g).

**Table 1:** Specific primers and probe for qPCR.

Gene name	Primer Sequences 5′-3′
PCV2-F	TGGGGCCACCTGGGTGTG
PCV2-R	GCCAAAAAAGGTACAGTTCCACC
PCV2-probe	FAM-AGCAAATGGGCTGCTAATTTTGCAGAC-TAMRA
GADPH-F (Zhang et al., 2021)	CCTTCCGTGTCCCTACTGCCAAC
GADPH-R (Zhang et al., 2021)	GACGCCTGCTTCACCACCTTCT
OASL-F (Li et al., 2017)	TCCCTGGGAAGAATGTGCAG
OASL-R (Li et al., 2017)	CCCTGGCAAGAGCATAGTGT
IFN-β-F	AGCACTGGCTGGAATGAAACCG
IFN-β-R	CTCCAGGTCATCCATCTGCCCA
TNF-α-F	ACCACGCTCTTCTGCCTACTGC
TNF-α-R	TCCCTCGGCTTTGACATTGGCTAC
IL-1β-F	CCCAAAAGTTACCCGAAGAGG
IL-1β-R	TCTGCTTGAGAGGTGCTGATG
PEDV-N-F	TCGTACTGAGGGTGTTTTCTGG
PEDV-N-R	CAACAATCTCAACTACGCTGGG

### 2.4. Detection of PCV2 replication in porcine intestinal cell lines

To test whether intestinal epithelial can support PCV2 replication, 50% monolayer of PK15 cells, IPI-FX cells, and IPEC-J2 cells were infected with PCV2d-LNHC at a multiplicity of infection (MOI) of 2 and were then cultured for 72 h at 37°C under 5% CO_2_. PCV2-infection-positive cells were detected with IPMA, as previously described (Huang et al., 2011).

To evaluate the proliferation kinetics of PCV2 in IPI-FX and IPEC-J2 cells, these two cell lines were grown to 50% confluence, infected with PCV2d-LNHC (MOI = 5), and then incubated at 37°C under 5% CO_2_. The cells and their supernatants were harvested together at 12, 24, 36, 48, 60, and 72 h post infection (hpi), and their viral titers were determined with IPMA, as previously described (Huang et al., 2011). The PCV2 reproduction kinetics curve was generated with the culture time as the abscissa and the viral titer as the ordinate. PCV2-infected IPI-FX cells were stained with an immunofluorescence assay (IFA) at 12, 24, 36, 48, 60, and 72 hpi, as described previously, with minor revisions (Huang et al., 2019). PCV2 was stained with the anti-PCV2 Rep monoclonal antibody (mAb) 3D1 and the anti-PCV2 Cap mAb 9H4, followed by Alexa-Fluor™-488-conugated goat anti-mouse IgG1 (Invitrogen) diluted 1:1000 in phosphate-buffered saline (PBS) and Alexa-Fluor™-594-conjugated goat anti-mouse IgG2b (Invitrogen) diluted 1:1000 in PBS. To visualize the cell nuclei, the cells were washed three times with PBS and stained with 4′,6′-diamidino-2-phenylindole (DAPI) for 10 min at room temperature. The stained cells were analyzed with fluorescence confocal microscopy (LSM 880 confocal laser scanning microscope with Airyscan; Zeiss, Germany). Cells were inoculated with strain PCV2d-LNHC at 0 hpi and used as the negative control.

### 2.5. Model of PCV2 and PEDV co-infection in IPI-FX cells

IPI-FX cells are often used to study the biological properties of PEDV *in vitro*. To establish a co-infection model *in vitro*, IPI-FX cells were infected with PCV2 and PEDV to study the effects of PCV2 infection on PEDV. IPI-FX cells grown to 50% confluence were infected with PCV2 (MOI = 1), cultured in the present of 2.5 μg/ml trypsin for 24 h at 37°C under 5% CO_2_, inoculated with PEDV (MOI = 1), and cultured for 24 h at 37°C under 5% CO_2_. The infected cells were then fixed with 4% (wt/vol) paraformaldehyde in PBS for 30 min at 4°C, washed three times with PBS, and permeabilized with 0.2% Triton X-100 for 20 min at room temperature. The cells were washed three more times with PBS, and then were blocked with 5% skim milk for 1 h at room temperature. The infected cells were stained with anti-PCV2 Cap mAb 9H4 or anti-PEDV N mAb 4H7 for 1 h at 37°C and then with Alexa-Fluor™-594-conjugated goat anti-mouse IgG2b (1:1000 in PBS; Invitrogen) or Alexa-Fluor™-488-conjugated goat anti-mouse IgG1 (1:1000 in PBS; Invitrogen) for 1 h at 37°C. To visualize the nuclei, the cells were stained with DAPI for 10 min at room temperature, and then washed three times with PBS. The stained cells were analyzed with fluorescence confocal microscopy (LSM 880 confocal laser scanning microscope with Airyscan).

### 2.6. Relative reverse transcription–qPCR (RT–qPCR)

Relative RT–qPCR was used to determine the expression of the PEDV nucleocapsid (N) gene and host cytokine mRNAs. The total RNA of the samples was extracted with the Simply P Total RNA Extraction Kit (Bioflux, Hangzhou, China), according to the manufacturer’s instructions. Equal amounts of total RNA (2 μg) were used to synthesize cDNA with PrimeScript RT Master Mix (Takara -Bio, Dalian,China). The 20 μl PCRs contained 10 μl of 2 × TB Green Premix Ex-Taq (Takara), 0.4 μl of primer mix (final concentration 200 nM), 1 μl of cDNA template, and 8.6 μl of nuclease-free water. The thermal cycling conditions were: 30 s at 95°C, followed by 40 cycles of denaturation at 95°C for 5 s and combined annealing and extension at 60°C for 30 s.

The specific primers for PEDV-N, interferon β (IFN-β), tumor necrosis factor α (TNF-α), interleukin 1β (IL1β), 2′,5′-oligoadenylate synthetase-like protein (OASL), and glyceraldehyde 3-phosphate dehydrogenase (GADPH; the endogenous control) used for RT–qPCR are listed in Table 1. The cycle threshold (CT) values and the differences in the CT values (ΔCT) for PEDV-N or host-cytokines genes were determined. The relative transcription levels of the these genes were calculated as the fold changes relative to the control with the 2^−ΔΔCT^ method (Livak and Schmittgen, 2001). The RT–qPCRs were performed with the QuantStudio™ 5 Real-Time PCR System(Applied Biosystems, Waltham, MA, United States), and the data were analyzed with the QuantStudio™ 5 Real-Time PCR System software.

### 2.7. Virus titration of PEDV

To determine the titers of PEDV in the IPI-FX cell supernatants, virus titration was performed on Vero E6 cells as previously described (Chua et al., 2008). Briefly, the supernatants of samples were collected and serially diluted 10-fold with DMEM contain 10 μg/ml trypsin. After that the diluted supernatants were inoculated on monolayers of Vero E6 cells and cells were incubated in DMEM contain 10 μg/ml trypsin for 4 days before observation to detect a cytopathic effect. The titers were calculated with the Reed–Muench method.

### 2.8. Western blotting

Western blotting was used to detect the PEDV N protein. The treated cells were lysed in NP40 lysis buffer (Beyotime, Shanghai, China) supplemented with Protease Inhibitor Cocktail (Sigma-Aldrich). Equal amounts of the extract were separated by SDS-PAGE, and the proteins were transferred to polyvinylidene difluoride (PVDF) membranes (Merck Millipore, MA, United States). After the membranes were blocked with PBS containing Tween 20 (PBST) and 5% skimmed milk powder, they were incubated with mouse anti-PEDV N mAb (diluted 1:10000) as the primary antibody and then with IRDye®-800CW-conjugated goat anti-mouse IgG (H + L) antibody (diluted 1:10000; Li-Cor Biosciences, Lincoln, NE, USA) as the secondary antibody. The blots were scanned with the Odyssey Infrared Imaging System (Li-Cor Biosciences). The mouse mAb directed against PEDV N was prepared and stored in our laboratory.

### 2.9. PCV2 pH tolerance test

Strain PCV2d-LNHC was propagated in PK15 cells and stored at −20°C. PCV2d-LNHC (250 μl) was diluted to 2.5 ml with PBS in a 15 ml centrifuge tube, and 13.5 μl of 5 M HCL was added to the tube to adjust the pH to 2.0. The samples were incubated at 37°C for the indicated times. After incubation, 16.0 μl of 5 M NaOH was added to the centrifuge tubes to adjust the pH to 7.2–7.4. The viral titers were quantified with virus titration based on IPMA, as described above.

### 2.10. Statistical analysis

All data are presented as the means ± standard deviations (SD) of three independent experiments and analyzed with Student’s *t* test in the GraphPad Prism 7.0 software (Graph-Pad Software, San Diego, California, United States).

## 3. Results

### 3.1. PCV2 Was mainly distributed in the jejunum and ileum

**Figure 1:**
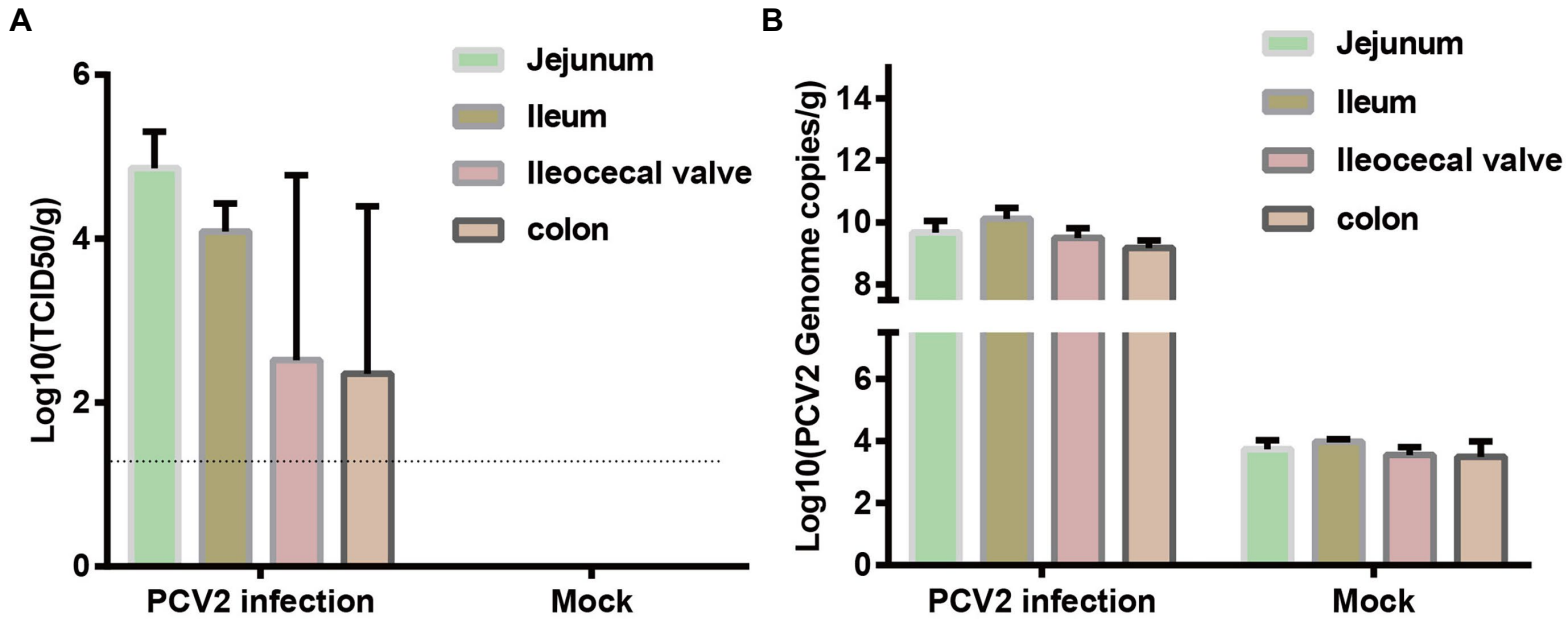
Distribution of PCV2 in different segments of porcine intestinal tissues. Three piglets in each group were infected with PCV2d-LNHC or medium (negative control). Samples from different intestinal segments from the six piglets were collected to detect the distribution of PCV2 at 28 dpi. **(A)** Virus titration was used to determine the titer of PCV2 in the intestinal tissues. The dotted line shows the detection limit, and the value under the dotted line is considered to be ‘undetected’. **(B)** Number of PCV2 genomes determined with qPCR.

The distribution of PCV2 in different intestinal segments was determined with virus titration (Figure 1A) and qPCR (Figure 1B). The titers of PCV2 in the jejunum, ileum, ileocecal valve, and colon were 10^4.86^, 10^4.09^, 10^2.52^, and 10^2.35^ TCID50/g, respectively. The qPCR results showed that the concentrations of PCV2 DNA in the jejunum, ileum, ileocecal valve, and colon were 5.88 × 10^9^, 1.63 × 10^10^, 3.24 × 10^9^, and 1.67 × 10^9^ copies/g, respectively. No PCV2 or its nucleic acid was detected in the intestinal tissues of the pigs in the control group. Therefore, PCV2 replicates in the intestinal tracts of infected piglets.

### 3.2. PCV2 Replicates in porcine epithelial cell lines

**Figure 2:**
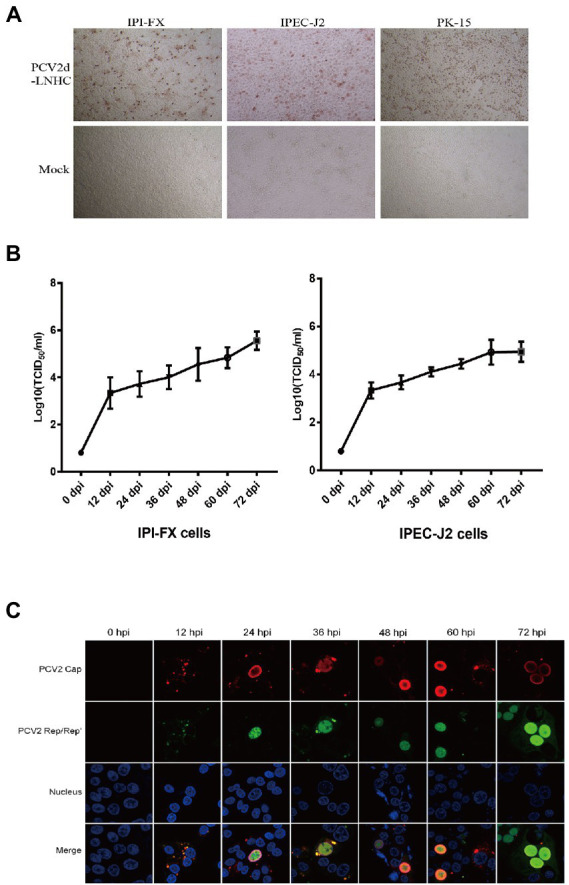
PCV2 replicates in IPI-FX and IPEC-J2 cells. IPI-FX and IPEC-J2 cells were inoculated with PCV2d-LNHC (MOI = 2). After culture for 72 h, the cells were fixed and stained with an IPMA **(A)**. IPI-FX and IPEC-J2 cells were inoculated with PCV2d-LNHC (MOI = 5) and then harvested and freeze–thawed at the indicated times to obtain viral stocks and the viral titers were determined. Growth curves were plotted with the viral titers thus determined **(B)**. IPI-FX cells were inoculated with PCV2d-LNHC. The cells were then fixed and stained with antibodies at the indicated time points, and immunofluorescence was observed with laser confocal microscopy **(C)**.

PCV2 was incubated with the IPI-FX and IPEC-J2 cells for 72 h. Viral infection was detected with IPMA, and the results showed that both the IPI-FX and IPEC-J2 cells could be infected with PCV2 (Figure 2A). Brown-stained positive cells were detected among the IPI-FX and IPEC-J2 cells as in the PK15 cells, whereas no specifically positive cells were detected in the mock-infected cells (Figure 2A). To evaluate the proliferation kinetics of PCV2 in IPI-FX and IPEC cells, the cells and their supernatants were harvested at 12, 24, 36, 48, 60, and 72 hpi, and the viral titers were measured in PK15 cells. The titers of PCV2 increased as the infection time increased, and the highest PCV2 titer in the IPI-FX cells line (10^5.5^ TCID_50_/ml) was higher than that in the IPEC-J2 cells (10^5.0^ TCID_50_/ml) (Figure 2B). These results show that PCV2 infects and replicates in small-intestinal cell lines.

IFA was used to assess the subcellular localization of the viral proteins in the IPI-FX cells at different times after infection (Figure 2C). The expression of the granular Cap (red signal) and Rep/Rep′ proteins (green signal) was detected in the cytoplasm at 12 hpi. Both the Cap and Rep/Rep′ proteins entered the nuclei at 24 hpi, although the Cap protein was more strongly distributed at the inner periphery of the nucleus. The most frequently observed phenomenon was the predominant distribution of the Cap and Rep/Rep′ proteins in the nuclei at 36–72 hpi. However, the distribution of the Cap protein signal in the cytoplasm was not commonly observed during late viral infection, unlike in PK15 cells, as described previously (Huang et al., 2019).

### 3.3. IPI-FX cells is amenable to PCV2 and PEDV co-infection

**Figure 3:**
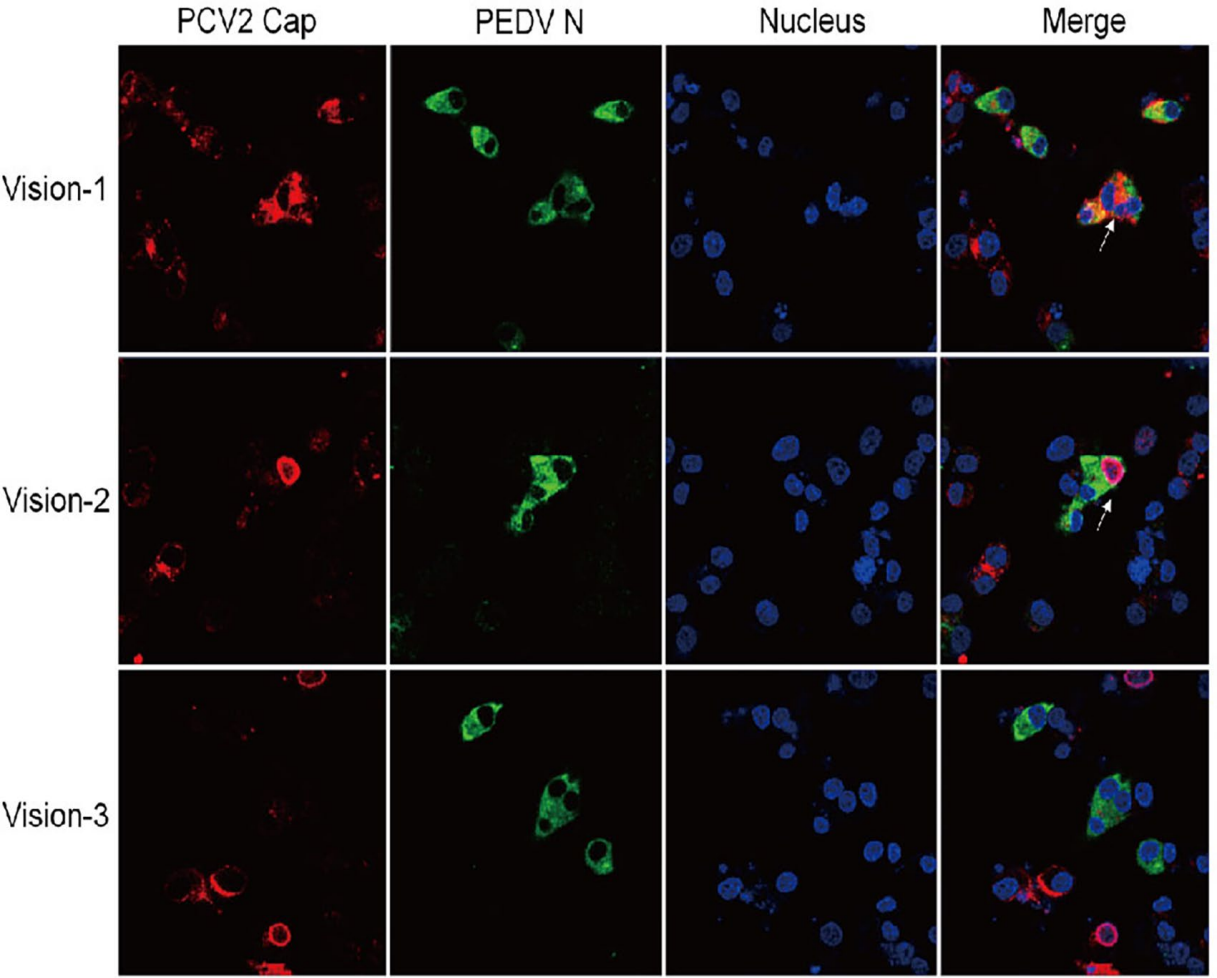
IPI-FX cells support the replication of both PEDV and PCV2 together. IPI-FX cells were inoculated with both PEDV and PCV2d-LNHC. At 72 h after inoculation, the cells were fixed, stained with an immunofluorescence assay (IFA), and observed with laser confocal microscopy. White arrows indicate cells infected with PEDV and PCV2.

To select a cell line amenable to co-infection with PCV2 and PEDV, two widely used porcine cell lines, IPI-FX and IPEC-J2, were inoculated with PEDV at MOI = 5. The IPI-FX cells were more sensitive than IPEC-J2 cells to PEDV/sx1a infection (data not shown). To assess whether PEDV and PCV2 could infect and replicate in the same cells, IPI-FX cells infected with PCV2 and PEDV were detected with IFA. The signals for PEDV N protein (green) and PCV2 Cap protein (red) were detected in different and the same IPI-FX cells in all randomly selected fields (Figure 3), indicating that PCV2 and PEDV can co-exist in or co-infect these cells.

### 3.4. PCV2 infection dose-dependently interferes with PEDV replication in IPI-FX cells

**Figure 4:**
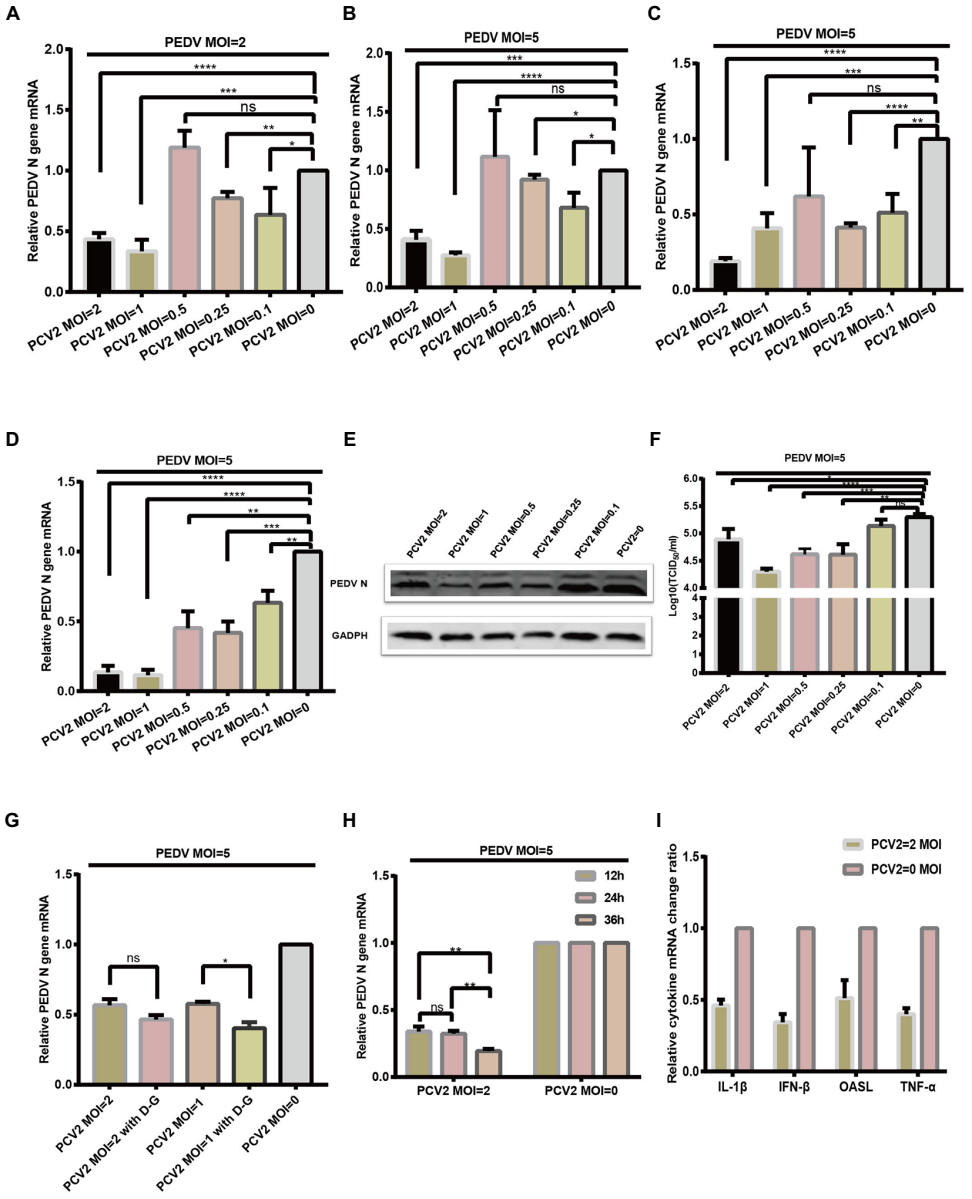
PCV2 reduces PEDV replication in IPI-FX cells. IPI-FX cells were inoculated PCV2d-LNHC at MOI = 2, 1, 0.5, 0.25, or 0.1 and incubated for 24 h. The same cells were then inoculated with PEDV (MOI = 2 or 5) and incubated for another 24 h. The cells were collected, the total RNA extracted, and the relative expression levels of nucleocapsid (N) mRNA in the PEDV–PCV2 dual-infected or PEDV-infected cells determined with RT–qPCR **(A,B)**. IPI-FX cells were inoculated PCV2a-LG or PCV2b-MDJ at MOI = 2, 1, 0.5, 0.25, or 0.1 and incubated for 24 h. The same cells were then inoculated with PEDV (MOI = 5) and incubated for another 24 h. The cells were collected, the total RNA extracted, and the relative expression levels of nucleocapsid (N) mRNA in the PEDV–PCV2 dual-infected or PEDV-infected cells determined with RT–qPCR **(C,D)**. IPI-FX cells were inoculated PCV2d-LNHC at MOI = 2, 1, 0.5, 0.25, or 0.1 and incubated for 24 h. The same cells were then inoculated with PEDV (MOI = 5) and incubated for another 24 h. After that, the cells were collected, lysed, and analyzed with western blotting using the antibodies indicated on the left. GADPH was loaded as the internal control **(E)**. IPI-FX cells were inoculated PCV2d-LNHC at MOI = 2, 1, 0.5, 0.25, or 0.1 and incubated for 24 h. The same cells were then inoculated with PEDV (MOI = 5) and incubated for another 24 h. The cells and supernatants were freeze–thawed to obtain viral stocks. The viral titers were determined with the Reed–Muench method **(F)**. IPI-FX cells were inoculated with PCV2d-LNHC (MOI = 2 or 1) and incubated for 24 h with or without d-glucosamine. The cells were then inoculated with PEDV (MOI = 5) and incubated for another 24 h. The cells were collected, the total RNA extracted, and the relative expression levels of nucleocapsid (N) mRNA in the PEDV–PCV2 dual-infected or PEDV-infected cells determined with RT–qPCR **(G)**. IPI-FX cells were inoculated with PCV2d-LNHC (MOI = 2) and incubated for the indicated times, and then with PEDV (MOI = 5) and incubated for another 24 h. The cells were collected, the total RNA extracted, and the relative expression levels of nucleocapsid (N) mRNA in the PEDV–PCV2 dual-infected or PEDV-infected cells determined with RT–qPCR **(H)**. IPI-FX cells were inoculated with PCV2d-LNHC (MOI = 2) and incubated for 24 h, and then with PEDV (MOI = 5) and incubated for another 24 h. The cells were collected, the total RNA extracted, and the relative expression levels of IL1β, IFN-β, OASL, and TNF-α mRNAs in the PEDV–PCV2 dual-infected and PEDV-infected cells determined with RT–qPCR using specific primers for IL1β, IFN-β, OASL, and TNF-α **(I)**. GADPH mRNA was used as the internal control for normalization **(A–I)**. Means ± standard deviations were calculated from three independent replicates of each experiments.

Having demonstrated that PCV2 and PEDV can co-infect IPI-FX cells, we next investigated the effect of PCV2 infection on PEDV replication. IPI-FX cells were inoculated with different doses (MOI = 2, 1, 0.5, 0.25, or 0.1) of PCV2d-LNHC and incubated for 24 h and then infected with PEDV (MOI = 2 or 5) and incubated for another 24 h. The PEDV N mRNA levels were quantified with RT–qPCR. As shown in Figures 4A,B, when the MOI of PEDV was 2, the mRNA levels of PEDV changed regularly after infection with different doses of PCV2: 56.5, 66.4, 22.7 and 36.5% inhibition, after inoculation with PCV2 (MOI = 2, 1, 0.25 and 0.1). when the MOI of PEDV was 5, the mRNA levels of PEDV N gene downregulated 59.3, 72.4, 7.8 and 31.8%, respectively after inoculation with different doses of PCV2d-LNHC. These results demonstrate that PCV2d-LNHC infection inhibited PEDV infection and replication in IPI-FX cells after sequential infection with PCV2 and PEDV. After sequential infection of IPI-FX cells with PCV2 and then PEDV, the inhibitory effect of PCV2 infection on PEDV was dose-dependent. PCV2a, PCV2b are also important epidemic genotypes of PCV2 in Asia. We want to know if PCV2a and PCV2b genotype can exert similar effect as PCV2d, so the PCV2a-LG strain and PCV2b-MDJ strain were selected to conduct sequential infection assay and detected with qPCR. As shown in Figures 4C,D, the infection of PCV2a and PCV2b also exerted inhibitory effect of sequentially PEDV infection in IPI-FX cells. Since PCV2d genotype is the main prevalent genotype, PCV2a, PCV2b and PCV2d strains showed similar effects on PEDV infection after sequential infection, so the PCV2d-LNHC strain was selected for the following experiments.

IPI-FX cells were also collected and analyzed with western blotting. When the infectious dose of PCV2 was MOI = 2, 1,0.5, 0.25 or 0.1 and that of PEDV was MOI = 5, the expression of PEDV N protein was decreased, respectively, compared with the single PEDV infection (Figure 4E). The sample supernatant was collected, and the titers of PEDV were determined (Figure 4F). When 1 MOI PCV2 and 5 MOI PEDV was successively inoculated on IPI-FX cells, the PEDV titer in the IPI-FX supernatant was 10^4.29^ TCID50/ml, which was 10 times less than that of the single PEDV infection (Figure 4F). When IPI-FX cells were successively infected with 2, 0.5 or 0.25 MOI PCV2 and 5 MOI PEDV, the viral titers of PEDV in samples were also significantly lower than that of single PEDV infection (Figure 4F).

We then examined whether promoting the replication of PCV2 inhibited the replication of PEDV. D-Glucosamine promotes the replication of PCV2 in PK15 cells (Yang et al., 2013). As shown in Figure 4G, the addition of d-glucosamine had a greater inhibitory effect on PEDV. We also found that as the PCV2 replication time in cells increased, the level of the PEDV infection decreased (Figure 4H).

We then examined whether the sequential infection of IPI-FX cells with PCV2 and PEDV affected The expression of The host’s cytokines. IPI-FX cells were infected with PCV2 (MOI = 2) and cultured for 24 h. they were then inoculated with PEDV (MOI = 5) cultured for a further 24 h. The mRNA levels of several cytokines were determined with RT–qPCR, and The results Are shown In Figure 4I. The expression of genes encoding IFN-β, TNF-α, and IL1β In The sequentially infected group decreased By 34.4, 40.0, and 46.1% respectively, compared with their expression In The group infected with PEDV alone.

### 3.5. PCV2 promotes PEDV replication In IPI-FX cells after simultaneous infection

**Figure 5:**
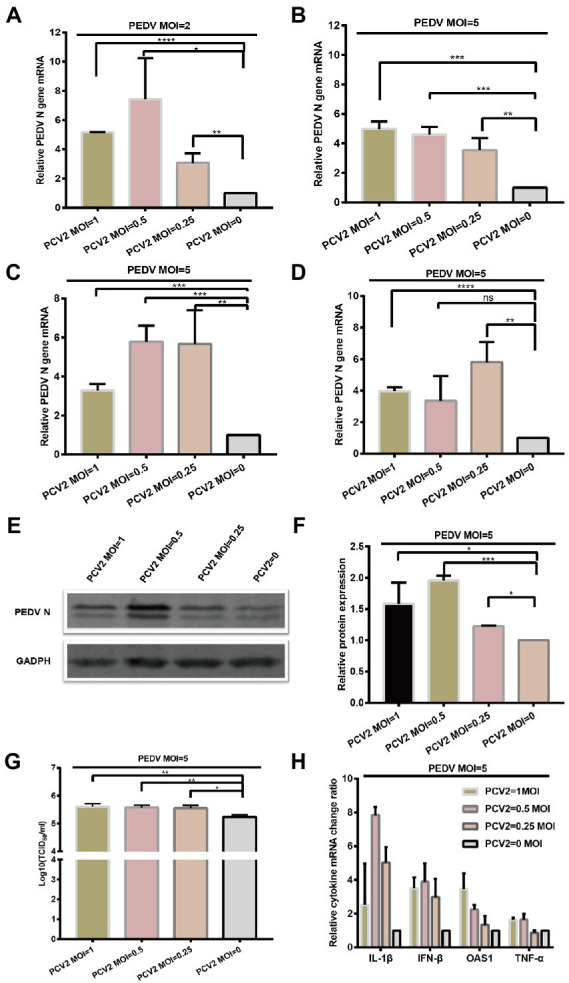
PCV2 promotes PEDV replication in IPI-FX cells. IPI-FX cells were inoculated with PEDV (MOI = 2 or 5) and simultaneously with PCV2d-LNHC (MOI = 1, 0.5, or 0.25). The cells were collected, the total RNA extracted, and the relative expression levels of nucleocapsid (N) mRNA in the PEDV–PCV2 simultaneously infected or PEDV-infected cells determined with RT–qPCR at 24 hpi **(A,B)**. IPI-FX cells were inoculated with PEDV (MOI = 5) and simultaneously with PCV2a-LG or PCV2b-MDJ (MOI = 1, 0.5, or 0.25). The cells were collected, the total RNA extracted, and the relative expression levels of nucleocapsid (N) mRNA in the PEDV–PCV2 simultaneously infected or PEDV-infected cells determined with RT–qPCR at 24 hpi **(C,D)**. IPI-FX cells were inoculated with PEDV (MOI = 5) and simultaneously with PCV2d-LNHC (MOI = 1, 0.5, or 0.25). At 24 hpi, the cells were collected, lysed, and analyzed with western blotting using the antibodies indicated on the left. GADPH was loaded as the internal control **(E)**. Western blotting results were quantified with ImageJ (National Institutes of Health, Bethesda, Maryland, United States) **(F)**. IPI-FX cells were inoculated with PEDV (MOI = 5) and simultaneously with PCV2d-LNHC (MOI = 1, 0.5, or 0.25). At 24 hpi, the cells and supernatants were freeze–thawed to obtain viral stocks. The viral titers were determined with the Reed–Muench method **(G)**. Relative expression levels of IL1β, IFN-β, OASL, and TNF-α mRNAs in the PEDV–PCV2 simultaneously infected and PEDV-infected cells were also determined **(H)**. GADPH mRNA was used as the internal control for normalization. Means ± standard deviations were calculated from three independent replicates of each experiment **(A–H)**.

Next, we wanted to investigate the effect of PCV2 on PEDV replication when PCV2 and PEDV were simultaneously inoculated in IPI-FX cells. In this case, IPI-FX cells were simultaneously inoculated with PEDV and PCV2d-LNHC and cultured for 24 h. Compared with PEDV infection alone, when the infectious dose of PCV2 was MOI = 1, 0.5, or 0.25 and the infectious dose of PEDV was MOI = 2, PEDV N mRNA increased by 517, 743, and 307%, respectively (Figure 5A). When the infectious dose of PCV2 remained unchanged and the infectious dose of PEDV was increased to MOI = 5, PEDV N mRNA also increased (Figure 5B). These results suggest that PCV2d-LNHC promotes the infection and replication of PEDV in IPI-FX cells when PCV2 and PEDV are co-infected simultaneously. Also, PCV2a-LG strain and PCV2b-MDJ strain were simultaneously inoculated on IPI-FX cells with PEDV. The results suggest PCV2a-LG strain and PCV2b-MDJ strain showed similar promotion effect of PEDV infection (Figures 5C,D).

As described above, PCV2d-LNHC strain was selected for western blotting and virus titration detection. The IPI-FX cells were also collected and analyzed with western blotting. When the infectious dose of PCV2 was MOI = 1, 0.5, or 0.25 and that of PEDV was MOI = 5, the expression of PEDV N protein increased by 158, 195%, or 122%, respectively, compared with the single PEDV infection (Figures 5E,F). The sample supernatant was collected, and the titer of PEDV determined (Figure 5G). When co-infected simultaneously with 1 MOI PCV2 and 5 MOI PEDV, the PEDV titer in the IPI-FX supernatant was 10^5.67^ TCID50/ml, which was 3.2 times higher than that of the single PEDV infection (Figure 5G). When 0.5 or 0.25 MOI PCV2 was simultaneously infected with 5 MOI PEDV, the viral titers of PEDV in samples were also significantly higher than that of single PEDV infection (Figure 5G).

Finally,the mRNA levels of several cytokines were determined, and showed that the transcription of *IFNB1*, *TNF*, *IL1B*, and *OASL* was higher in the co-infection group than in the singly infected group (Figure 5H), which may have been the consequence of increased PEDV replication.

### 3.6. PCV2 tolerates pH 2.0

**Figure 6:**
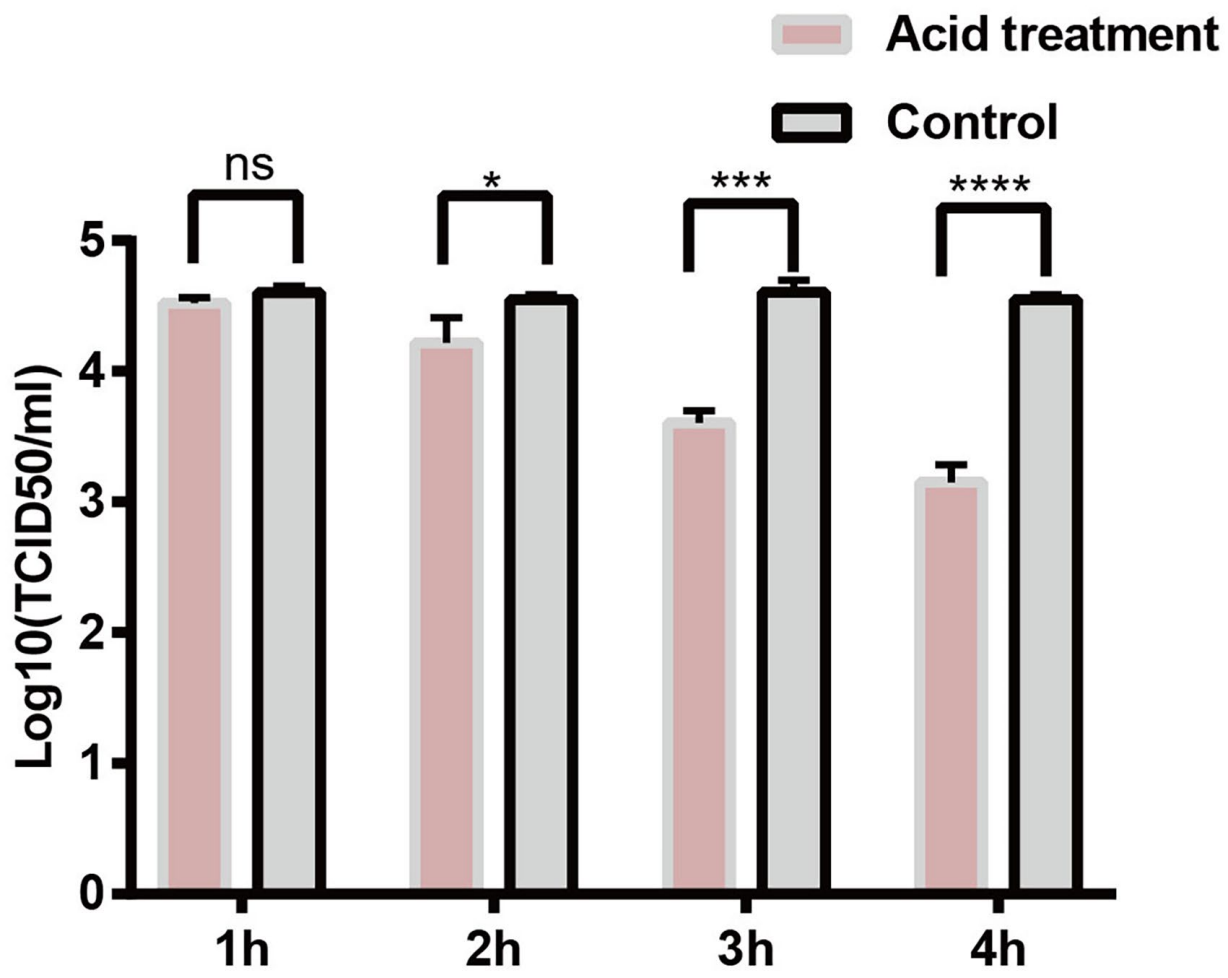
PCV2 tolerates environmental pH 2.0. PK15 cells were inoculated with PCV2d-LNHC at pH 2.0 and incubated for 1–4 h at 37°C. The viral titers in the acid-treated PCV2-infected and mock-PCV2-infected cells were determined with virus titration.

PCV2d-LNHC was incubated at pH 2.0 for 1–4 h at 37°C to detect the acid tolerance of the virus. The experimental results showed that the ability of PCV2 to infect PK15 cells was not affected by the gastric acid environment (pH 2.0) *in vitro* for 1 h (Figure 6). About 1/10 of PCV2 still infected PK15 cells after 4 h at pH 2.0 *in vitro* (Figure 6). These results suggest that PCV2 remains infectious after incubating at pH 2.0 for up to 4 h.

## 4. Discussion

PCV2 is the smallest known DNA virus that can infect mammals. PCV2 infections can be traced back to the 1960s (Jacobsen et al., 2009). Its tissue tropism in susceptible animals includes the lymphoid system, lung, spleen, kidney, and other organs and tissues. Therefore, infected pigs show a variety of clinical symptoms. PCV-associated enteritis mainly manifests as diarrhea and granulomatous enteritis, and the typical microscopic pathological changes are lymphopenia, increased histiocytes, and multinucleated giant cells in Peyer’s patches.

Several studies have reported that the peak in PCV2 fecal shedding occurs 2–3 weeks after PCV2 infection (Patterson et al., 2011). In the present study, large amounts of PCV2 were isolated from the jejunums and ileums of PCV2-infected pigs, suggesting that PCV2 excretion through the feces is closely related to PCV2 infection of the small-intestinal tissues. Therefore, the intestinal tissues of pigs are probably sites of PCV2 replication. We preliminarily tested whether PCV2 tolerates the gastric acid in the intestinal tract by simulating the gastric acid environment *in vitro*. The experimental results showed that the ability of PCV2 to infect PK15 cells was not affected by the gastric acid environment (pH 2.0) *in vitro* for 1 h. About 1/10 of PCV2 still infected PK15 cells after 4 h at pH 2.0 *in vitro* (the time required for gastric emptying is generally 4 h). We infer that PCV2 can withstand the acidic environment of the stomach, and therefore infects small-intestinal cells *via* the digestive tract. The intestinal tissues of piglets were also collected and sent for immunohistochemical staining. Unfortunately, the IHC staining results for three piglets from PCV2 infection group were all negative, although the virus titration and qPCR results for these piglets were all positive. Therefore, it is still unclear whether the jejunal or ileal epithelial cells or the distributed immune cells of PCV2-infected pigs support PCV2 replication. Further research is required.

It has been reported that PCV2 can replicate in the IPEC cells, and it has been observed to move along microtubules in the IPEC cells (Yan et al., 2014). We found that PCV2 infected IPI-FX and IPEC-J2 cells with similar propagation kinetics as when it infected PK15 cells, but the viral titers were lower than in PK15 cells. However, the detection of PCV2 Cap and Rep/Rep′ proteins showed that PCV2 replicated differently in IPI-FX cells than in PK15 cells. In IPI-FX cells, the Cap and Rep/Rep′ proteins entered the cell nuclei later than in the PK15 cells. Although there was no obvious diffusion of the Cap protein signal during the period of observation, the fluorescent signals for Cap and Rep/Rep′ accumulated in the nuclei and became increasingly stronger, indicating that the Cap and Rep proteins of PCV2 were continuously expressed.

PCV2 and PEDV infect piglets before and after weaning (Gerber et al., 2012; Meng, 2013; Choudhury et al., 2016). Epidemiological surveys have shown that PCV2 and PEDV co-infections are common, with a co-infection rate of 3.47–57.89% (Guo et al., 2020; Ma et al., 2021). Several studies have detected PCV2 in the Peyer’s patches of the small intestine, and have shown that it affects the immune system of the small intestine. In the present study, we detected large numbers of infectious PCV2 in small-intestinal tissues, which were also the main site of PEDV infection. Therefore, we tested whether PCV2 also infects small-intestinal epithelial cells, like PEDV.

Several laboratories have reported that the IPEC-J2 cells supports the infection and replication of PEDV with low efficiency. However, the IPEC-J2 cells stored in our laboratory was incapable of supporting infection and replication of PEDV/SX1a strain, with or without the addition of trypsin. The IPI-FX cell line is a subclone of the IPI-2I cell line, which was isolated and established from the ileal tissue of a 2-day-old piglet (Wang et al., 2019). Fortunately, IPI-FX cells support PEDV and PCV2 co-infection, so we used them to investigate the effect of PCV2 infection on PEDV replication *in vitro*.

Both PEDV and PCV2 can infect IPI-FX cells. However, the replication of virus in the cells requires the host’s organelles and corresponding enzymes, and this could negatively affect the replication of the other virus (Shih and Liu, 2020; Xu et al., 2020; Romeo et al., 2021). Another study demonstrated this phenomenon when PCV2 and classical swine fever virus (CSFV) co-infected PK15 and ST cells (Zhou et al., 2015). We found similar results when PCV2 and PEDV sequentially infected IPI-FX cells (Figures 4A,B), indicating that PCV2 infection inhibits subsequent PEDV replication. We used various methods to promote PCV2 replication to confirm the occupancy effect of PCV2, and found that either the addition d-glucosamine or increasing the incubation time of PCV2 inhibited the replication of PEDV.Besides, a strategy involving the simultaneous inoculation of both viruses was tested, and showed that PCV2 infection promoted PEDV replication. These results suggest when PCV2 and PEDV infect IPI-FX cells in different ways, PCV2 can exert inhibitory or promotional effects. Therefore, we speculate that the infection of pigs with these two viruses at different times and in different sequences may have different consequences. Further research in pigs is required to clarify these phenomena.

The innate immune system is the first line of defense against microbial infection. After a virus infects a cell, virus-specific nucleic acids and proteins are recognized, and the innate immune pathway is activated. Molecules such as IRF3 are often required to enter the nucleus to initiate the transcription and translation of proteins with antiviral activity, such as IFN-β, TNF-α, IL1β, and OASL. The transcripts of these cytokines correlated positively with the amount of PEDV progeny virus at 24 h after PEDV and PCV2 co-infection. This result suggests that PEDV infection of IPI-FX cells activates the innate immune pathway, so the transcription of the corresponding cytokines is upregulated as PEDV reproduction progresses. However, the mRNAs of IFN-β, TNF-α, IL1β, and OASL were downregulated in cells sequentially infected with PCV2 and PEDV. Possible explanations are: (1) PCV2 infection inhibits the innate immune system in IPI-FX cells, because although PEDV activates the innate immune system of the cells, the inhibitory effect of PCV2 prevails; (2) PCV2 infects the cells before PEDV and inhibits PEDV replication, resulting in the observed downregulation of cytokines.

In conclusion, large amounts of PCV2 were isolated from the jejunums and ileums of PCV2-infected piglets, demonstrating that the small-intestinal tissues support PCV2 replication. IPI-FX cells also supported the replication of PCV2, and a co-infection model of PCV2 and PEDV was successfully established *in vitro*. After the sequential infection of PCV2 and PEDV, the replication of PEDV was largely inhibited, whereas after the simultaneous infection of IPI-FX cells with PCV2 and PEDV, the replication of PEDV was promoted. These co-infection experiments *in vitro* confirmed that PCV2 affects the replication of PEDV in small-intestinal epithelial cells, and lay a foundation for the study and identification of the co-infection mechanisms of PCV2 and PEDV.

## Data availability statement

The original contributions presented in the study are included in the article/supplementary material, further inquiries can be directed to the corresponding authors.

## Ethics statement

This study was ethically approved by the Experimentation and Laboratory Animal Welfare Committee of the Harbin Veterinary Research Institute (HVRI) of the Chinese Academy of Agricultural Sciences (CAAS) with the approval code of 210512-10. The Harbin Veterinary Research Institute’s and China guidelines for the Care and Use of Laboratory Animals were followed.

## Author contributions

HZ and HS contributed equally to this study by performing the experiments, analyzing data, and drafting the manuscript. YW performed animal infection, sample collection, and data analysis. DS, MC, JbL, and JhL performed the experiment. LL prepared and provided the reagents exclusively for this research. CL, LF, and LH conceived the study, performed animal experiments, and edited and finalized the manuscript. All authors contributed to the article and approved the submitted version.

## Funding

This work was supported by the National Key Research and Development Program of China (2022YFD1800300).

## Conflict of interest

The authors declare that the research was conducted in the absence of any commercial or financial relationships that could be construed as a potential conflict of interest.

## Publisher’s note

All claims expressed in this article are solely those of the authors and do not necessarily represent those of their affiliated organizations, or those of the publisher, the editors and the reviewers. Any product that may be evaluated in this article, or claim that may be made by its manufacturer, is not guaranteed or endorsed by the publisher.

## Abbreviations

PCV2: porcine circovirus type 2
PEDV: porcine epidemic diarrhea virus
TCID50: tissue culture infectious dose
qPCR: real-time quantitative PCR
IFN-β: interferon β
TNF-α: tumor necrosis factor α
IL1β: interleukin 1β
OASL: 2′,5′-oligoadenylate synthetase-like protein
PCVAD: porcine-circovirus-associated diseases
PMWS: post-weaning multisystemic wasting syndrome
DMEM: Dulbecco’s Modified Eagle’s Medium
FBS: fetal bovine serum
ASFV: African swine fever virus
PRRSV: Porcine reproductive and respiratory syndrome virus
PRV: pseudorabies virus
MOI: multiplicity of infection; hpi, hours post infection
IFA: immunofluorescence assay
PBS: phosphate-buffered saline; DAPI, 4′,6′-diamidino-2-phenylindole
GADPH: glyceraldehyde 3-phosphate dehydrogenase
PVDF: polyvinylidene difluoride
PBST: PBS containing Tween 20
IPMA: immunoperoxidase monolayer assay
D-G: D-glucosamine
